# First-in-human study of the PARP/tankyrase inhibitor E7449 in patients with advanced solid tumours and evaluation of a novel drug-response predictor

**DOI:** 10.1038/s41416-020-0916-5

**Published:** 2020-06-11

**Authors:** Ruth Plummer, Divyanshu Dua, Nicola Cresti, Yvette Drew, Peter Stephens, Marie Foegh, Steen Knudsen, Pallavi Sachdev, Bipin M. Mistry, Vaishali Dixit, Sharon McGonigle, Nancy Hall, Mark Matijevic, Shannon McGrath, Debashis Sarker

**Affiliations:** 1 Northern Institute for Cancer Care, Freeman Hospital and Newcastle University, Newcastle upon Tyne, UK; 2 The Canberra Hospital, Canberra, Australia; 3 Northern Centre for Cancer Care, The Newcastle upon Tyne Hospitals NHS Foundation Trust, Newcastle upon Tyne, UK; 4 Royal Devon and Exeter NHS Foundation Trust, Exeter, Devon UK; 5 Oncology Venture US Inc, Cambridge, MA USA; 6 Oncology Venture A/S, Hørsholm, Denmark; 7 Eisai Inc., Woodcliff Lake, NJ USA; 8 Eisai Inc, Andover, MA USA; 9 King’s College London and Guy’s and St Thomas’ NHS Foundation Trust, London, UK

**Keywords:** Cancer therapy, Cancer

## Abstract

**Background:**

This phase 1 study examined the safety, maximum-tolerated dose (MTD) and antitumour activity of E7449, a novel PARP 1/2 and tankyrase 1/2 inhibitor.

**Methods:**

E7449 was orally administered once daily in 28-day cycles to patients with advanced solid tumours (50–800-mg doses). Archival tumour samples from consenting patients were evaluated for the expression of 414 genes in a biomarker panel (2X-121 drug-response predictor [DRP]) found to be predictive of the response to E7449 in cell lines.

**Results:**

Forty-one patients were enrolled (13 pancreatic, 5 ovarian, 4 each with breast, lung or colorectal cancer and 11 with other tumour types). The most common grade ≥3 treatment-related adverse event was fatigue (*n* = 7, 17.1%). Five patients experienced a dose-limiting toxicity (fatigue, *n* = 4, 800 mg; anaphylaxis, *n* = 1, 600 mg) for an MTD of 600 mg. E7449 exhibited antitumour activity in solid tumours, including 2 partial responses (PRs), and stable disease (SD) in 13 patients, which was durable (>23 weeks) for 8 patients. In 13 patients, the 2X-121 DRP identified those achieving PR and durable SD. E7449 showed good tolerability, promising antitumour activity and significant concentration-dependent PARP inhibition following 50–800-mg oral dosing.

**Conclusion:**

The results support further clinical investigation of E7449 and its associated biomarker 2X-121 DRP.

**Clinical trial registration:**

www.ClinicalTrials.gov code: NCT01618136.

## Background

Polyadenosine diphosphate (ADP)-ribose polymerases (PARPs) are a family of highly conserved enzymes involved in a variety of important cellular processes, including DNA damage repair.^1–3^ Two PARP family members (PARP1 and PARP2) are nuclear enzymes that, upon activation in response to DNA strand breaks, synthesise and transfer long branches of poly (ADP ribose) (PAR) onto DNA-associated proteins via poly(ADP ribosyl)ation.^2–4^ This results in a negatively charged environment, which facilitates recruitment of repair machinery and accelerates DNA damage repair.^4,5^ Because many anticancer therapies act by inducing DNA damage in tumour cells, investigating the therapeutic potential of PARP inhibitors has generated considerable interest.^6,7^

PARP inhibitors exert cytotoxic effects through the inhibition of the catalytic activity of PARP1/PARP2 by trapping PARP–DNA complexes, thereby preventing DNA replication and transcription.^8^ Potent and specific inhibitors of PARP have been shown to sensitise cancer cells to the DNA-damaging effects of cytotoxic chemotherapy and radiotherapy.^9^ One of the key features of the DNA repair pathway is redundancy or the capacity for alternative proteins or molecular pathways to compensate for specific repair deficiencies. In this pathway, there is increasing interest in the genetic principle of “synthetic lethality,” whereby deficiency of a sole DNA repair component renders tumour cells highly sensitive to specific inhibition of a redundant pathway that would otherwise be nonessential.^9^ PARP inhibitors have demonstrated activity as single agents in tumours deficient in DNA repair (i.e., *BRCA*-deficient cancers), and have increased the cytotoxic effects of certain chemotherapies when administered in combination regimens.^9^ Three PARP inhibitors (olaparib, niraparib and rucaparib) are approved for the treatment of women with recurrent advanced ovarian cancer and deleterious germline or somatic *BRCA* mutations.^10–15^ The US Food and Drug Administration approved olaparib in the first-line maintenance setting for patients with *BRCA*-mutated advanced ovarian cancer following the significant benefit in progression-free survival seen in the randomised, placebo-controlled, phase 3 SOLO1 trial.^10,16^ The PARP inhibitors olaparib and niraparib are also indicated for the maintenance treatment of women with platinum-sensitive, recurrent ovarian, fallopian tube or primary peritoneal cancer; niraparib can be used regardless of *BRCA*-mutation status, whereas olaparib is indicated for patients with *BRCA* mutations.^10,14–16^

E7449 (also known as 2X-121, herein referred to as E7449) is an orally bioavailable, small-molecule inhibitor of the enzymatic activity of PARP1 and PARP2 (inhibitory concentration at half-maximal effect [IC_50_] of 1.0 and 1.2 nmol/L, respectively), which traps PARP1 on DNA via dose-dependent binding to chromatin.^17^ E7449 also inhibits tankyrase 1 and 2, which are important regulators of the canonical Wnt/β-catenin signalling pathway involved in promoting tumorigenesis. In a Wnt1 preclinical model, synergistic antitumour effects were observed when E7449 was used in combination with the mitogen-activated protein kinase kinase inhibitor E6210.^17^ In addition, E7449-mediated tankyrase inhibition results in a significant increase in axin2 levels, and a concomitant reduction in active and total β-catenin in human colon cancer SW480 cells.^17^ E7449 demonstrated single-agent antitumour activity in several *BRCA*-deficient xenograft models, as well as potentiation of the efficacy of radiotherapy and chemotherapy in both in vitro and in vivo models, respectively.^17,18^ In this first-in-human study, we report the results of an open-label, multicentre, phase 1 trial of single-agent E7449 in patients with advanced malignancies. In addition, we report a novel tumour-agnostic molecular biomarker, 2X-121 drug-response predictor [DRP], developed and tested post study, to identify patients as responders or non-responders to therapy.

## Methods

### Study design, treatment schedule and assessments

This was an open-label, multicentre, phase 1 study of single-agent E7449 (ClinicalTrials.gov: NCT01618136). The primary endpoint was to determine the maximum-tolerated dose (MTD) of E7449. Secondary endpoints included assessments of safety and tolerability, pharmacokinetic (PK) profile (including the effect of a high-fat meal on E7449 bioavailability), pharmacodynamic (PD) effects (including inhibition of PARP in peripheral blood mononuclear cells [PBMCs] and effect on DNA damage), PK/PD relationships, serum biomarker analysis and preliminary antitumour activity. The study was conducted in full accordance with the principles of the World Medical Association Declaration of Helsinki, the International Conference on Harmonisation and all applicable local guidelines and regulations on good clinical practice. All patients provided written informed consent.

The study included a 3 + 3 dose-escalation phase in which sequential cohorts of 3–6 patients were administered increasing oral doses of E7449 (starting at 50 mg), administered once daily in 28-day cycles. To further assess the safety and tolerability of E7499 at the MTD, and to determine the recommended phase 2 dose, an expansion cohort of 9–12 additional response-evaluable patients was planned. Patients received study drug until disease progression, development of unacceptable toxicity or withdrawal of consent.

The MTD was defined as the highest dose of E7449 at which ≤1 of 6 patients demonstrated a dose-limiting toxicity (DLT). DLTs were assessed according to Common Terminology Criteria for Adverse Events, version 4.03, and were defined as any grade 3 or worse haematologic or nonhaematologic toxicity occurring during cycle 1 of the dose-escalation phase, and considered related to E7449 by the investigator. DLTs included any grade 4 neutropenia occurring for ≥7 days or grade 3 neutropenia with fever (>38.5 °C in axilla), grade 4 or grade 3 thrombocytopenia with bleeding or lasting >7 days, grade 3 fatigue or a 2-point decline in Eastern Cooperative Oncology Group (ECOG) performance status persisting for over 7 days, persistent grade 3 or 4 nausea, vomiting or diarrhoea, despite maximal medical therapy, grade 3 or worse nonhaematologic laboratory abnormalities requiring hospitalisation and E7449-related adverse events (AEs) occurring during cycle 1, resulting in the administration of <75% of the planned E7449 dosage.

The effect of food on E7449 exposure was evaluated in cycle 1 for patients included in the expansion cohort. These patients fasted overnight on day 7, after which, they were randomised to receive E7449 with or without a high-fat breakfast. Fed patients were administered E7449 immediately after consuming a meal. Patients were then crossed over to the alternate food regimen on day 15.

### Patient eligibility

Eligible patients were ≥18 years of age with histologic and/or cytologic confirmation of advanced or metastatic solid tumours or B-cell lymphomas progressing after treatment with approved therapies, or for which no standard therapies exist. Patients could have measurable or non-measurable disease by Response Evaluation Criteria in Solid Tumors, version 1.1, but only patients with measurable disease were permitted to enter the extension phase of the study. All eligible patients had adequate cardiac, bone marrow, renal and liver functions, a life expectancy of ≥3 months, an ECOG performance status of 0–2 and a left ventricular ejection fraction of >50%. Patients with brain metastases were eligible if they had undergone complete surgical excision or stereotactic radiosurgery or radiotherapy, and had no radiographic evidence of brain disease recurrence/progression, were asymptomatic and had discontinued corticosteroid treatment ≥30 days prior to dosing.

Key exclusion criteria included prior exposure to E7449, leptomeningeal or brain metastases (except for that as mentioned in the inclusion criteria), use of strong-cytochrome P450 inhibitors or inducers, any major surgery or anticancer treatment ≤4 weeks prior to dosing, active infection requiring systemic therapy, prolongation of heart rate-corrected QT interval to >480 ms when electrolyte balance was normal or had any uncontrolled endocrine disease necessitating relevant changes in medication within the last month or hospital admission within the previous 3 months.

### Safety

Safety was measured by monitoring and recording all AEs, including all Common Terminology Criteria for Adverse Events grades, and serious AEs (SAEs), clinical laboratory test results, vital sign measurements and electrocardiogram and physical examination findings.

### Pharmacokinetic and pharmacodynamic assessments

For the dose-escalation cohorts, blood samples for pharmacokinetic analyses were collected at 0, 0.5, 1, 2, 3, 4, 6, 8, 10, 12, 24 and 48 h after a single dose of E7449 administered on day −2, and again at the same time points at steady state on day 15 of cycle 1 (excluding the 48-h sample). Blood samples were also collected immediately before the dose on day 7 of cycle 1, days 1 and 15 of cycle 2 and on day 1 of cycle 3, and beyond. All urine produced during the time intervals pre-dose, 0–2 h, 2–4 h, 4–10 h and 10–24 h following the single dose on day –2, and after multiple doses on cycle 1, day 15, was collected.

Validated liquid chromatography coupled with tandem mass spectrometry methods was used to determine human plasma and urine concentrations of E7449 (XenoBiotic Laboratories Inc., [WuXi AppTec], Plainsboro, NJ, USA). PK parameters were calculated from the E7449 plasma concentration–time profiles and urine excretion data using noncompartmental methods with the Phoenix WinNonlin version 6.2.

For the dose-escalation phase of the study, blood samples for pharmacodynamic analyses were collected at 0, 2, 4, 8 and 24 h after single-dose administration of E7449 on day –2 and cycle 1, day 15, and at 0 h and 4 h post dose on cycle 1, day 1 and cycle 1, day 7. Inhibition of PARP activity was measured by quantifying PAR in PBMCs using an enzyme-linked immunosorbent assay (HT PARP in vivo Pharmacodynamic Assay II kit). PAR levels in PBMCs and the percentage change from baseline were summarised by dose and nominal sampling time point.

For the food-effect expansion cohort, blood samples for the pharmacokinetic analyses were collected prior to the E7449 dose and then 1, 2, 3, 4, 8, 12 and 24 h after E7449 dosing on days 7 and 15 of cycle 1, when E7449 was at steady state.

### Pharmacokinetic/pharmacodynamic modelling

The E7449 plasma concentration-versus-time and PAR inhibition data in PBMCs were subjected to PK/PD modelling using a 2-stage approach. In the first stage, the mean plasma concentration-versus-time curves of E7449 from all doses were simultaneously fitted to a 2-compartment pharmacokinetic model with first-order absorption and -elimination from the central compartment. In the second stage, the final parameters from the pharmacokinetic model were fixed for sequential PK/PD modelling.

The PD measurement utilised in the PK/PD model was the percentage inhibition of the formation of PAR, a marker for PARP activity,^19^ which was calculated as % inhibition = (Response/Baseline) × 100, where baseline was defined as the PAR response at time 0 on day –2. The percent change in PAR from baseline (%PAR) over time, as a function of E7449 plasma concentrations, was fitted to a sigmoidal inhibitory indirect-response PK/PD model, where E7449 inhibited the rate of formation (*k*_in_) of PAR.^20^

The PK/PD model equation is shown below:

$$\frac{{d\left( {{\mathrm{\% }}{\mathrm{PAR}}} \right)}}{{dt}} = k_{{\mathrm{in}}} \ast \left( {\frac{{I_{{\mathrm{max}}} \ast C^{g_{{\mathrm{am}}}}}}{{{\mathrm{IC}}_{50}^{g_{{\mathrm{am}}}} + C^{g_{{\mathrm{am}}}}}}} \right) - k_{{\mathrm{out}}} \ast {\mathrm{PAR}}$$

where *C* is the concentration of E7449 in the central compartment, *I*_max_ is the maximal inhibitory effect (percent inhibition from baseline), which was assumed to be 100%, IC_50_ is the E7449 concentration [ng/mL] at half-maximal effect and *g*_am_ is the sigmoidicity factor (Hill coefficient).

The model parameters from the PK/PD analysis were then used to simulate the expected PAR responses of additional dosing regimens of E7449. All PK/PD modelling and subsequent response simulations were performed using Phoenix WinNonlin^®^, version 7.

### Efficacy

Tumour assessments (computed tomography, magnetic resonance imaging, photographs and bone scans, as suitable for tumour type), based on Response Evaluation Criteria in Solid Tumors version 1.1, were performed at screening and subsequently every 8 weeks until disease progression. Investigator assessments of complete or partial response were confirmed ≥4 weeks after initial assessment.

### Serum biomarker analysis

To investigate the correlation of biomarkers and the biological activity of single-agent E7449, blood samples were obtained from 27 patients in the dose-escalation cohort at specified time points, processed for serum, aliquoted and stored frozen. Two pre-dose, multiple post-dose and off-treatment time points were included. Serum samples were tested for concentrations of soluble vascular endothelial growth factor receptor (VEGFR)2, VEGFR3 and soluble interleukin-2 receptor α (sIL-2Rα/CD25), using the bead-based Human Soluble Cytokine Receptor Panel assay kit. The two baseline samples (days −3 and −2 prior to dosing) were averaged, and the mean was used to calculate percent change at each of the post-dosing time points.

### Tumour biomarker analysis

Seventy-four cell lines were treated with E7449, and the IC_50_ was calculated after 8 days of treatment (Supplementary Table 1). Matching baseline transcriptome and growth inhibition data were available for 61 of the 74 cell lines, and were used to develop a DRP as previously described.^21,22^ Briefly, IC_50_ values in the 61 cell lines were correlated to gene-expression levels in the same cell lines; 172 genes with a Pearson correlation above 0.25 were retained as potential markers of sensitivity, while 242 genes with a Pearson correlation below −0.25 were retained as potential markers of resistance. The 414 genes were searched in PubMed for known associations to DNA damage response or Wnt/β-catenin pathways. The 111 pathway-associated genes are shown in Supplementary Table 2. Archival biopsies, in the form of formalin-fixed paraffin-embedded blocks and slides, were obtained from patients in the trial. Only those samples that yielded RNA of sufficient quantity and quality were evaluated for gene expression. The expression of the 414 genes in the 2X-121 DRP was analysed via a custom Affymetrix U133_Plus2 microarray according to the manufacturer’s instructions. The 2X-121 DRP was applied in a blinded manner following a prespecified analysis plan. The difference between the means of the genes that were positively correlated and those negatively correlated to response was used as a single prediction score for each patient, after normalisation to a range of 0–100.

### Statistical analysis

As a first-in-human phase 1 clinical trial, no formal sample-size calculation or hypothesis testing was undertaken. Analysis of biomarker predictions was performed using a 1-sided Pearson correlation test and a 1-sided log-rank test for time-to-event data according to a prespecified analysis plan.

## Results

### Study population

The study was conducted between January 31, 2012 and July 14, 2015, and enrolled 41 patients from 2 sites in the United Kingdom. All enrolled patients received 1 of 6 dose levels (50 mg, 100 mg, 200 mg, 400 mg, 600 mg and 800 mg) of single-agent E7449 in the dose-escalation phase of the study. Of the 33 patients who completed the first cycle of treatment, 32 patients continued to receive the study drug, and were included in the extension phase of the study. Most patients completed the dose-escalation and extension phases of the study (80% [33/41] and 84% [27/32], respectively); 21 patients received the MTD (600 mg), 13 of whom then enrolled as part of the food-effect cohort in the expansion phase.

Demographic and baseline characteristics of the patients are summarised in Table 1. Most patients had an ECOG performance status of 0 (22.0%) or 1 (58.5%); 78% had received ≥2 prior anticancer therapies. The most common primary tumour sites were pancreas (*n* = 13), ovary (*n* = 5), breast (*n* = 4), lung (*n* = 4), colorectal (*n* = 4) and pleura (*n* = 3). Although *BRCA* testing was not mandated by the protocol, a total of 6 patients had germline *BRCA* mutations confirmed by the investigator (ovarian cancer, *n* = 3, breast cancer, *n* = 2 and pancreatic cancer, *n* = 1).

**Table 1:** Patient baseline characteristics (safety analysis set).

Characteristic	Patients, *n* (%) (*N* = 41)
Median age, years (range)	65.0 (24, 76)
Sex	
Male	22 (53.7)
Female	19 (46.3)
Race	
White	40 (97.6)
Asian	1 (2.4)
Eastern Cooperative Oncology Group performance status	
0	9 (22.0)
1	24 (58.5)
2	1 (2.4)
Missing	7 (17.1)
Number of prior anticancer therapies	
0	1 (2.4)
1	7 (17.1)
2	14 (34.1)
3	7 (17.1)
4	5 (12.2)
5	5 (12.2)
6	1 (2.4)
Location of primary tumour	
Adrenal glands	1 (2.4)
Breast^a^	4 (9.8)
Gallbladder and extrahepatic bile ducts	1 (2.4)
Kidney	1 (2.4)
Large intestine (excluding the appendix)	4 (9.8)
Lung and bronchus	4 (9.8)
Oesophagus	1 (2.4)
Ovary^b^	5 (12.2)
Pancreas^c^	13 (31.7)
Pleura	3 (7.3)
Retroperitoneum and peritoneum	1 (2.4)
Skin	1 (2.4)
Small intestine	2 (4.9)

^a^Two patients had *BRCA* mutation.

^b^Three patients had *BRCA* mutation.

^c^One patient had *BRCA* mutation.

### Safety

DLTs were observed in 5 of the 25 evaluable patients (Table 2). One DLT occurred in the 600-mg dose cohort (grade 3 anaphylactic reaction) and 4 in the 800-mg dose cohort (fatigue [grade 3, *n* = 1; grade 2, *n* = 3], leading to administration of <75% of the planned E7449 dose). An E7449 dose of 600 mg, administered orally and once daily, was defined as the MTD, and is the recommended phase 2 dose.

**Table 2:** Summary of exposure and dose-limiting toxicities (safety analysis set).

E7449 dose cohort (*n)*	Median number of cycles received (range)	Evaluable patients, *n*	Patients with dose-limiting toxicity, *n* (%)	Dose-limiting-toxicity description
50 mg (*n* = 3)	6 (1, 8)	3	0	—
100 mg (*n* = 3)	2 (2, 14)	3	0	—
200 mg (*n* = 4)	3 (1, 4)	4	0	—
400 mg (*n* = 4)	5 (0, 10)	3	0	—
600 mg^a^ (*n* = 8)	2 (0, 13)	6	1 (16.7)	Grade 3 anaphylactic reaction
800 mg (*n* = 6)	2 (0, 11)	6	4 (66.7)	Grade 3 fatigue (*n* = 1) Grade 2 fatigue (*n* = 3)

^a^Selected as the maximum-tolerated dose.

Across dose cohorts, the mean number of E7449 treatment cycles was 3.8 (median, 2; range, <1–14), and the median duration of E7449 treatment was 57 days (range, 1–392). Grade 3 AEs were reported in 27 patients (65.9%), with fatigue being the most common (17.1% [7/41]). One grade 4 AE (hypokalaemia, not treatment-related) was observed in 1 patient in the 200-mg dose cohort. No grade 5 AEs were reported. Nonfatal SAEs were reported in 58.5% (24/41) of patients.

Thirty-nine patients (95.1%) in the dose-escalation cohort had treatment-related AEs, with the most common being fatigue (63%), chromaturia (49%), nausea (34%), diarrhoea (29%), maculopapular rash (27%), photosensitivity reaction (24%) and decreased appetite (24%) (Table 3). Treatment-related grade 3 fatigue occurred in 4 patients (600 mg, *n* = 2; 800 mg, *n* = 2). E7449 treatment was discontinued in 17% (7/41) of patients due to AEs (50 mg, *n* = 1; 600 mg, *n* = 4; 800 mg, *n* = 2). Fatigue (*n* = 3) was the most common AE leading to treatment discontinuation.

**Table 3:** Most common treatment-related adverse events occurring in >5% of patients overall (any Common Terminology Criteria for Adverse Events grade; safety analysis set).

Preferred term, %^a^	E7449 dose cohort, mg						
	50 (*n* = 3)	100 (*n* = 3)	200 (*n* = 4)	400 (*n* = 4)	600^b^ (*n* = 21)	800 (*n* = 6)	Total (*N* = 41)
Fatigue	33.3	66.7	0	75.0	66.7	100.0	63.4
Chromaturia	0	0	0	50.0	66.7	66.7	48.8
Nausea	33.3	33.3	50.0	25.0	33.3	33.3	34.1
Diarrhoea	33.3	33.3	0	50.0	23.8	50.0	29.3
Maculopapular rash	0	33.3	25.0	25.0	33.3	16.7	26.8
Decreased appetite	33.3	0	0	25.0	23.8	50.0	24.4
Photosensitivity reaction	33.3	33.3	0	25.0	23.8	33.3	24.4
Vomiting	33.3	0	25.0	0	14.3	33.3	17.1
Depression	0	33.3	25.0	50.0	4.8	16.7	14.6
Periorbital oedema	0	0	0	25.0	19.0	0	12.2
Pruritus	0	33.3	0	0	19.0	0	12.2
Skin hyperpigmentation	0	33.3	0	0	9.5	33.3	12.2
Increased blood alkaline phosphatase	0	0	0	25.0	14.3	0	9.8
Constipation	33.3	33.3	0	0	9.5	0	9.8
Dry skin	0	33.3	0	0	4.8	33.3	9.8
Abdominal pain	0	33.3	0	25.0	0	16.7	7.3
Anaemia	33.3	0	0	0	9.5	0	7.3
Dizziness	0	0	0	0	4.8	33.3	7.3
Dysgeusia	0	0	0	0	14.3	0	7.3
Onycholysis	0	0	0	25.0	9.5	0	7.3
Erythematous rash	0	0	0	0	9.5	16.7	7.3

^a^Adverse events were coded using Medical Dictionary for Regulatory Activities version 17.1 and graded using Common Terminology Criteria for Adverse Events version 4.03.

^b^Maximum-tolerated-dose cohort, *n* = 8; food-effect cohort, *n* = 13.

Treatment-emergent AEs associated with skin rash events were reported by 41.5% (17/41) of patients who received ≥100-mg doses of E7449 (47.6% in the 600-mg dose cohort and 66.7% in the 800-mg dose cohort). Preferred AE terms associated with rash included maculopapular rash (*n* = 11), erythematous rash (*n* = 4), acneiform dermatitis (*n* = 2), blister (*n* = 1) and rash (*n* = 1). None of the reported skin rash events were considered serious, and all were of grade 1 or 2 severity, with the exception of one grade 3 erythematous rash event in the 600-mg dose cohort. Because E7449 treatment increased photosensitivity, patients were advised to avoid sun exposure, use sunscreen, wear protective clothing and to consult a dermatologist in the event of any rash occurring. In addition, treatment-emergent chromaturia was experienced by 51.2% (21/41) of patients (50.0% in the 400-mg, 66.7% in the 600-mg and 83.3% in the 800-mg dose cohorts), and all events of chromaturia were grade 1.

### Pharmacokinetics

E7449 PK parameters following a single dose and at steady state are summarised in Table 4. E7449 was rapidly absorbed with the *t*_max_ at steady state ranging from 0.5 to 4 h across dose cohorts. The elimination half-life, *t*_1/2_, was approximately 8 h, and the accumulation ratio, *R*_ac_, was less than 1.2-fold across the range of doses, indicative of no accumulation of E7449 at steady state. Less than 1.5% of the drug dose was excreted unchanged in urine. E7449 exposure (*C*_max_ and AUC) following oral administration was increased roughly proportionally from 50 mg to 800 mg following single or multiple doses. At the 600-mg dose, consumption of a high-fat meal delayed E7449 absorption, as indicated by a shift in *t*_max_ by 2 h, and a reduction in *C*_max_ by 60%. However, overall exposure, as measured by AUC (0–24 h or 0-t) increased to ~10% (Supplementary Table 3).

**Table 4:** E7449 pharmacokinetic parameters (pharmacokinetic analysis set).

Day –2 (single dose)						
Dose	50 mg	100 mg	200 mg	400 mg	600 mg	800 mg
*n*	3	3	4	4	8	6
*C*_max_ (ng/mL)	265 (99.8)	284 (112)	996 (676)	999 (619)	2250 (1330)	4430 (2470)
*t*_max_ (h)	2.00 (0.58, 2.03)	2.05 (1.08, 3.00)	3.10 (1.03, 4.18)	2.04 (1.03, 4.05)	2.12 (0.50, 24.3)	0.79 (0.50, 2.00)
AUC_(0–24)_ (ng·h/mL)	768 (*n* = 1)	879 (104)	3670 (1170)	4690 (2400)	7930 (4990)	11,300 (3230)
AUC_(0-t)_ (ng·h/mL)	603 (213)	879 (104)	3780 (1180)	4800 (2450)	8120 (4990)	11,500 (3370)
AUC_(0-inf)_ (ng·h/mL)	770 (*n* = 1)	895 (99.8)	3320 (1790) (*n* = 2)	4800 (3000) (*n* = 3)	10,700 (5040) (*n* = 4)	12,900 (3350) (*n* = 4)
*t*_1/2_ (h)	3.42 (*n* = 1)	6.35 (3.36)	7.08 (1.16) (*n* = 2)	8.62 (0.54) (*n* = 3)	7.17 (2.98) (*n* = 4)	9.45 (2.42) (*n* = 4)
*CL*/*F* (L/h)	64.9 (*n* = 1)	113 (12.9)	70.7 (38.3) (*n* = 2)	104 (50.1) (*n* = 3)	64.1 (23.8) (*n* = 4)	64.7 (13.4) (*n* = 4)
*V*_z_/*F* (L)	321 (*n* = 1)	1070 (687)	690 (273) (*n* = 2)	1320 (689) (*n* = 3)	741 (496) (*n* = 4)	864 (230) (*n* = 4)
*A*_e_ (mg)	0.11 (0.06) (*n* = 2)	0.47 (*n* = 1)	0.70 (0.60) (*n* = 3)	2.77 (1.82)	2.90 (0.51)	4.00 (1.73)
*f*_e_ (%)	0.22 (0.13) (*n* = 2)	0.47 (*n* = 1)	0.34 (0.29) (*n* = 3)	0.69 (0.45)	0.48 (0.09)	0.50 (0.22)
*CL*_R_ (L/h)	– (*n* = 0)	0.5 (*n* = 1)	0.2 (0.15) (*n* = 3)	0.6 (0.34)	5.0 (13.0)	0.4 (0.12)
*Cycle 1, day 15 (multiple once-daily doses), steady state*						

*n*	3	3	3	3	6	5
*C*_max_ (ng/mL)	264 (227)	404 (175)	1430 (1080)	1130 (1030)	2230 (1570)	4120 (2160)
*t*_max_ (h)	1.00 (0.5, 2.00)	3.00 (1.00, 4.00)	1.00 (1.00, 2.00)	4.00 (0.5, 4.00)	1.14 (0.50, 4.05)	0.50 (0.0, 1.97)
AUC_(0–24)_ (ng·h/mL)	714 (193)	1100 (244)	3690 (2230)	4730 (2730)	7900 (4730)	12,400 (5230)
AUC_(0-inf)_ (ng·h/mL)	634 (*n* = 1)	1150 (266)	2670 (1510) (*n* = 2)	3240 (820) (*n* = 2)	9660 (4550) (*n* = 4)	12,000 (5910) (*n* = 4)
*t*_1/2_ (h)	9.04 (*n* = 1)	8.92 (2.50)	9.19 (4.83) (*n* = 2)	4.16 (1.72) (*n* = 2)	5.05 (1.76) (*n* = 4)	6.44 (1.56) (*n* = 4)
*C*_av,ss_ (ng/mL)	29.7 (8.03)	45.8 (10.2)	154 (93.1)	197 (114)	329 (198)	516 (218)
*CL*_ss_ /*F* (L/h)	73.1 (17.1)	94.2 (22.9)	73.3 (50.4)	103 (50.5)	133 (139)	67.1 (28.2)
*R*_ac_	1.19 (*n* = 1)	1.19 (0.11)	1.22 (0.22) (n = 2)	1.03 (0.04) (*n* = 2)	1.05 (0.05) (*n* = 4)	1.09 (0.06) (*n* = 4)
*A*_e_ (mg)	0.19 (0.00) (*n* = 2)	0.70 (*n* = 1)	1.11 (0.33)	2.05 (1.09)	2.25 (1.28)	4.70 (2.91)
*f*_e_ (%)	0.39 (0.01) (*n* = 2)	0.70 (*n* = 1)	0.55 (0.17)	0.51 (0.27)	0.38 (0.21)	0.68 (0.47)
*CL*_R_ (L/h)	0.3 (0.01) (*n* = 2)	0.5 (*n* = 1)	0.5 (0.4)	0.4 (0.14)	0.3 (0.12)	0.5 (0.34)

Data are the mean (standard deviation) except for *t*_max_.

For *t*_max_, median (minimum – maximum) is shown.

*A*_e_, amount of drug dose excreted in urine; AUC_(0–24)_, area under the concentration–time curve from time zero (pre-dose) to 24 h post dose; AUC_(0-t)_, area under the concentration–time curve from time zero (pre-dose) to the time of the last quantifiable concentration; AUC_(0-inf)_, area under the concentration–time curve from time zero (pre-dose) extrapolated to infinite time; *C*_av,ss_, average steady-state concentration during multiple-dose administration; *C*_max_, maximum observed concentration; *CL/F*, apparent total clearance following oral administration; *CL*_ss_/*F*, apparent total clearance at steady state following oral administration; *CL*_R_, renal clearance; *f*_e_, cumulative fraction of drug dose excreted/recovered in urine; *R*_ac_, accumulation ratio; *t*_max_, time at which the highest drug concentration occurs; *t*_1/2_, terminal elimination phase half-life; *V*_z_/*F*, apparent volume of distribution at the terminal phase.

### Pharmacodynamics

**Fig. 1:**
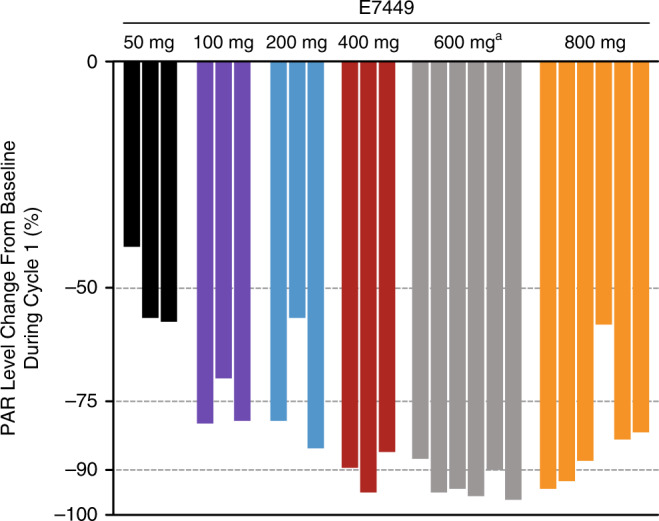
Maximum percentage of inhibition of PARP by patient and E7449 dose during cycle 1 (any time point). ^a^All patients in the 600-mg cohort exceeded the maximum % PARP inhibition measurement at 1 or more time points. Maximum inhibition was calculated when the measured PAR in peripheral blood mononuclear cells was below the assay lower detection limit. The measured value was then replaced by the assay lower detection limit. PAR, polyadenosine diphosphate–ribose; PARP, polyadenosine diphosphate–ribose polymerases.

Dose-dependent inhibition of PARP activity, as measured by the percentage change of PAR levels in PBMCs, was observed with E7449 treatment. Maximal inhibition was achieved at the MTD (600 mg once daily) (Fig. 1). At the MTD, the population mean frequency of PARP inhibition was approximately 90% at steady state (Fig. 1). PARP inhibition was sustained with a ≥ 70% decrease in PAR levels at 24-h post-dose time points (cycle 1, days 1, 7, 8 and 16 [at 0 h for each]). An increased effect of E7449 on PAR levels was observed with increased exposure to E7449 up to and including the 600-mg dose (Supplementary Fig. 1A). PAR levels remained suppressed over the 24-h dosing interval even though E7449 concentrations decreased rapidly (Supplementary Fig. 1B). In the food-effect cohorts with (fed and unfed) patients treated at the MTD, PARP inhibition was sustained during treatment with E7449 up to 24 h post dose. The maximal decrease in PAR levels occurred at 2–4 h after administration of E7449 in the fasted cohort, and 4–8 h post treatment in the fed cohort, with up to 90% inhibition in PAR levels from baseline (Supplementary Fig. 2).

### Pharmacokinetic/pharmacodynamic modelling

The fitted PK model curves and observed PK data for the six E7449 dose levels are shown in Supplementary Fig. 3A. The absorption rate constant (*k*_a_) was estimated to be 0.82 h^−1^ with a first-order elimination rate constant (*k*_e_) of 0.51 h^−1^. The apparent volume of distribution for the central compartment (V/F) was estimated to be 149.7 L, and the distribution rate constants between the central and the peripheral compartment were estimated to be 0.21 h^−1^ (*k*_12_) and 0.27 h^−1^ (*k*_21_). The compartmental PK parameters were estimated with reasonable confidence (% coefficient of variation range, 10–47%) (Supplementary Table 4).

The sigmoidal indirect-response PK/PD model fits the time course of target modulation reasonably well, with an estimated IC_50_ of 0.2 µg/mL PK (Supplementary Fig. 3B). The model tended to overpredict the percent inhibition of PAR at the lowest dose on both day 1 and day 15; however, the PK/PD model fits the higher-dose data reasonably well. The PD parameter estimates and their coefficients of variance are shown in Supplementary Table 4. The final PK/PD model was used to simulate different E7449 dosing regimens that may show sustained inhibition of PAR formation. The simulations indicate that a twice-daily E7449 dosing regimen is needed for sustained inhibition of the PARP enzyme. For example, simulations showed that a E7449 200-mg twice-daily or a 300-mg twice-daily dose showed a sustained inhibition of the target. However, the PAR response following the same dose given once a day (i.e., 400-mg or 600-mg once daily), returned to baseline faster than the twice-daily regimen.

### Efficacy

The objective response rate (ORR) was 4.9% (2/41) with 2 patients with ovarian cancer (1 each in the 600-mg and 800-mg dose cohorts) achieving a partial response. Thirteen (31.7%) of the 41 patients had the best overall response of stable disease with a median duration of 162 days (52–262 days). Of the 35 patients who were evaluated for the best overall response, 13 patients, including 3 patients with previously treated ovarian cancer (2 patients with partial response and 1 patient with stable disease), had a decrease in target lesion size (Supplementary Fig. 4). The ORR in patients with germline-mutated *BRCA* was 16.7% (1/6). Of the 6 patients with known *BRCA* mutations, stable disease was observed in 2 patients (pancreatic and high-grade serous ovarian tumour), partial response in 1 patient (high-grade serous ovarian tumours) and progressive disease in 3 patients (2 breast tumours and 1 ovarian tumour). None of the *BRCA-*mutated patients (*n* = 6) in this study had prior treatment with a PARP inhibitor, whereas two-thirds (4/6: ovarian, *n* = 3; breast, *n* = 1) had prior platinum exposure—most of whom were platinum-sensitive, and one was resistant, at the time of study enrolment (Supplementary Table 5).

### Serum biomarker analysis

Assessment of serum biomarker concentrations over time demonstrated that E7449 conferred an effect on sIL-2Rα concentrations, although a dose-dependent relationship between sIL-2Rα was not observed. Increased sIL-2Rα was observed from 48 h onwards and in all dose groups, with up to 150% change above baseline recorded (Supplementary Fig. 5A). A range of endogenous variability of sIL-2Rα concentrations was determined using serum samples collected within a 2-week time period from 10 healthy controls. The endogenous fluctuation of sIL-2Rα levels was determined to be +/−20% from baseline (Supplementary Fig. 5B). Thus, the observed increases in sIL-2Rα of up to 150% change from baseline appear to be E7449-dependent. No significant changes in serum concentrations from baseline were observed for sVEGFR2 and sVEGFR3 (data not shown).

### Tumour biomarker analysis

**Fig. 2:**
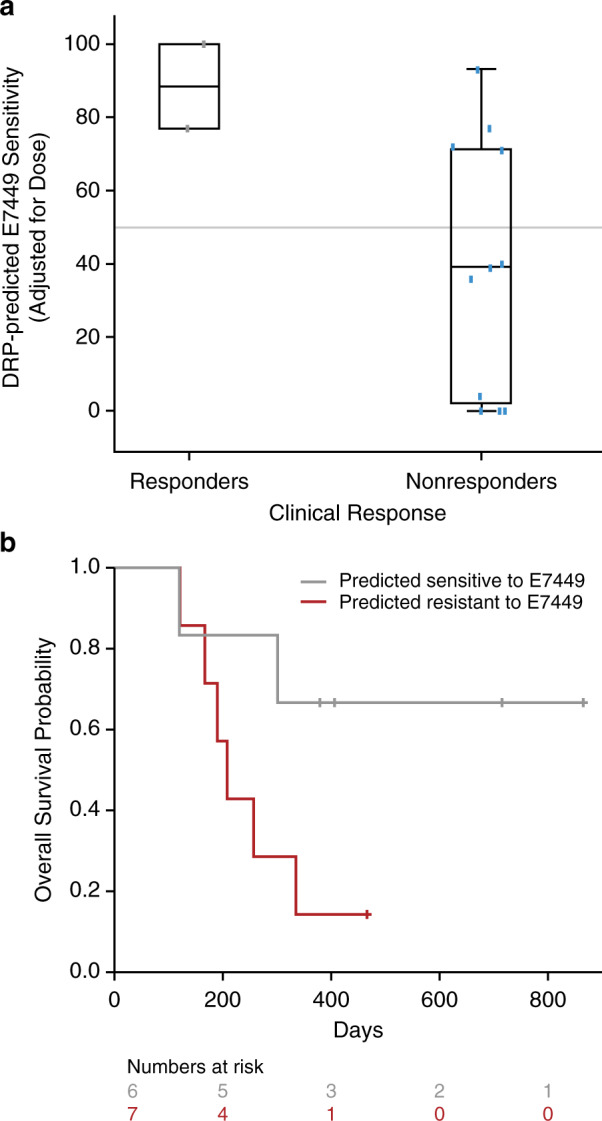
Biomarker predicted tumour sensitivity to E7449 versus clinical outcome (ORR). The prediction score was corrected for differences in dose by multiplying with the dose (50–800 mg) and dividing by 518. The grey mid-line shows the prespecified cut-off score of 50 used to divide patients into sensitive and not sensitive (**a**). Overall survival of the two groups of patients predicted sensitive or resistant (**b**).

A novel, tumour-agnostic, molecular biomarker, 2X-121 DRP, was developed to identify responders and non-responders using 61 cell lines as previously described.^21,22^ In these tumour cell lines, 414 genes were found to predict response to E7449: 172 genes were found to be positively correlated with sensitivity to E7449, and 242 genes were negatively correlated. Among these 414 genes predictive of the response to E7449, 111 were found to be associated in the literature with DNA damage response or Wnt/β-catenin pathways (Supplementary Table 2). Tumour biopsies from 16 patients in the trial were analysed, and the RNA samples from 13 of these patients, which were of sufficient quantity and quality, were evaluated for the expression of the 414 predictive genes used in the 2X-121 DRP (via a custom Affymetrix microarray). The 2X-121 DRP was applied to the gene-expression profiles obtained from tumour biopsies of these 13 patients and, using a prespecified DRP cut-off sensitivity score of 50, was used to predict sensitivity to E7449. The resultant DRP score correctly identified the 2 partial responders as highly sensitive (Fig. 2a). A non-responder whose 2X-121 DRP results (a score of 93) predicted the patient to be highly sensitive to E7449 showed extended progression-free survival (no progression by the last assessment at 321 days and alive at the last follow-up assessment at 406 days). The Pearson correlation between dose-adjusted prediction scores and changes in baseline tumour diameter was −0.43 (*P* = 0.07). Based on the DRP score and the prespecified cut-off of 50, 6 patients were predicted to be sensitive to E7449 treatment, and 7 patients were predicted to be not sensitive. The group of patients predicted to be sensitive, compared with those predicted to be not sensitive to E7449 treatment, did have a longer median time to progression (296 vs. 155 days, respectively; HR = 0.29; *P* = 0.06) and median overall survival (>800 days vs. 208 days, respectively; HR = 0.26; *P* = 0.04, Fig. 2b).

To determine whether the DRP is prognostic or predictive, we tested it in a published dataset of triple-negative breast cancer, which has previously been used for developing prognostic signatures.^23^ The hazard ratio in event-free survival between 32 patients predicted to be E7449-sensitive and 32 patients predicted to be E7449-resistant is 1.0, leading to the conclusion that the signature is likely not prognostic.

## Discussion

This phase 1 study evaluated the MTD, safety and tolerability, PK/PD profile and preliminary activity of single-agent E7449 in patients with advanced solid tumours. The study met its primary endpoint, and the MTD and recommended phase 2 dose of E7449 monotherapy was determined to be 600 mg, administered orally once daily.

PARP inhibitors are generally well tolerated as single agents. The most commonly reported AEs associated with the 3 approved PARP inhibitors are fatigue, nausea and vomiting, and haematologic AEs such as anaemia and thrombocytopenia.^24–26^ Consistent with these class effects, the most common E7449-related AE observed in this study was fatigue. Unexpectedly, E7449-related AEs included maculopapular rash (33.3% at the MTD), photosensitivity (23.8% at the MTD) and chromaturia (66.7% at the MTD), which have not been reported previously in clinical trials of other PARP inhibitors. Although rash appeared to be the result of E7449-related phototoxicity, its incidence was minimised through incorporation of sunlight-prevention measures. When skin rash appeared, it was improved with drug interruption. Chromaturia also appeared to be an E7449-specific event, resulting from generation of an E7449-derived metabolite that appeared green to the eye.

E7449-related haematological toxicity was observed infrequently in this study. At the MTD, grade 1 thrombocytopenia was observed in 1 patient (1/21, 5%), and anaemia (≤grade 2) was observed in 4 patients (4/21, 19%). The mean number of cycles was 3.8 (minimum, maximum: 0, 14), which may account for the limited number of treatment-related anaemia events observed in this study. As haematologic toxicity may serve as a barrier to combination strategies with chemotherapy,^24^ the lack of higher-grade haematologic toxicities with E7449 may facilitate the evaluation of combination regimens, which may be needed to treat advanced malignancies.

Following single or multiple doses, moderately rapid absorption of E7449 was observed. Although food reduced the absorption rate (as *C*_max_ declined by 60% and *t*_max_ increased by 2 h), overall absorption was minimally impacted with a modest 10% increase in AUC. PARP inhibition with E7449 was dose-dependent, with maximal inhibition of PARP activity at the E7449 MTD. In addition, sustained inhibition of PARP activity was achieved, and maximal inhibition of PARP activity correlated with peak plasma concentrations of E7449.

Veliparib and talazoparib are 2 additional oral PARP1/PARP2 inhibitors in clinical development that have demonstrated single-agent antitumour activity in both *BRCA*-positive and *BRCA*–wild-type tumours.^27–29^ In this study, E7449 monotherapy demonstrated preliminary antitumour activity in patients with advanced solid tumours, irrespective of *BRCA* status.

Compared to other licensed or investigational PARP inhibitors, the preclinical profile of E7449 most closely resembles that of olaparib and niraparib.^17,26^ Olaparib, niraparib and E7449 have all demonstrated trapping of PARP1 on DNA, resulting in augmented cytotoxicity.^17,30^

Using an indirect-response model, the relationship between E7449 plasma concentration and PARP inhibition was established. The PK/PD relationship provided a useful tool to evaluate additional dosing regimens, including the opportunity to define the optimal dosing strategy for E7449. PK/PD simulations suggest that twice-daily dosing may provide an improved profile of sustained PARP inhibition over 24 h, especially at lower doses. Twice-daily dosing regimens have been adopted for other PARP inhibitors, including olaparib^10^ and veliparib.^27^ Dosing schedules for these particular PARP inhibitors are based primarily on PK parameters, whereas in this study, we provide both PK and PD data in support of E7449 twice-daily dosing.

In preclinical studies, E7449 at a dose of 100 mg/kg, resulted in sustained PARP inhibition in tumours, similar to published reports for olaparib and niraparib.^17,31,32^ In addition to inhibition of PARP1/PARP2, E7449 is a robust inhibitor of the telomere-associated PARP enzymes tankyrase 1/2.^17^ While no PD assessments of the tankyrase-inhibitory activity of E7449 were clinically available at the time of this trial, preclinical studies indicate that E7449 inhibits tankyrase 1/2 with IC_50_ values from 50 to 120 nmol/L.^17^ In contrast, the IC_50_ values for tankyrase inhibition by olaparib, niraparib and veliparib are 5–20-fold higher.^17^ Therefore, the greater inhibition of tankyrase 1/2 by E7449 differentiates it from other PARP inhibitors, and might lead to distinct clinical opportunities for this agent. In particular, patients with tumours showing aberrant activation of the Wnt/β-catenin pathways (e.g., colorectal and lung cancer) may benefit from tankyrase-targeted therapy.^33,34^

Inhibition of DNA repair with PARP inhibitors is an effective cytotoxic strategy when used in patients with tumours that are deficient in *BRCA1* and *BRCA2*.^27^ In this study, 50% (3/6) of patients with *BRCA* mutations showed a response of either partial response (*n* = 1, high-grade serous ovarian tumour) or stable disease (*n* = 2, pancreatic tumour and high-grade serous ovarian tumour), highlighting the preliminary activity of E7449 in patients with *BRCA* mutations. As PARP inhibition has proven to be a highly effective therapeutic approach in the treatment of ovarian cancers that have an underlying defect in DNA repair mechanisms, conducting randomised, phase 2 trials to evaluate the efficacy of E7449 in tumours with mutations in DNA repair genes may be useful.

Since PARP1 is known to influence both tumour vasculature and gene transcription in immune cells,^35,36^ exploratory assessment of serum immune-related and angiogenesis biomarker concentrations over time was assessed. A significant increase from baseline in sIL-2Rα in patients receiving E7449 was observed. It has been suggested that sIL-2Rα levels may serve as a marker of regulatory T-cell (Treg) number and/or function.^37,38^ Interestingly, it has been shown that PARP1 regulates Treg function via FOXp3 signalling.^39,40^ Experiments in preclinical models demonstrate that direct inhibition of both PARP1 and PARP1-knockout mice shows increases in the numbers of Tregs in the periphery.^41,42^ Although our biomarker observations of increased serum sIL-2Rα with E7449 dosing only represent a surrogate of potential Treg activity, the results are consistent with preclinical observations. While additional studies examining the effects of PARP inhibitors on Treg populations are still needed, these preliminary data suggest that investigation into a combination approach using a PARP inhibitor and a Treg-targeting agent should be considered.

The 2X-121 DRP is a step towards realising the promise of personalised medicine—by using patients’ tumour gene-expression profiles, it successfully predicted response to treatment with the PARP inhibitor, E7449. The 2X-121 DRP successfully combined microarray and drug-sensitivity data from cell lines to predict the response. While the number of samples used for testing the novel 2X-121 DRP biomarker is very limited, this drug-specific biomarker approach has previously been applied to other chemotherapies, and has demonstrated its predictive value.^21,22^ Moreover, additional studies, including two ongoing phase 2 clinical trials (NCT03878849 and NCT03562832), wherein the 2X-121 DRP biomarker will be employed prospectively, will be used to further validate these results.

In conclusion, the safety, tolerability and preliminary efficacy of once-daily E7449 600 mg in patients with advanced solid tumours seems promising, encouraging further clinical evaluation of this drug. To this end, two phase 2 clinical trials investigating the efficacy of E7449 (aka, 2X-121) in patients selected by the 2X-121 DRP are currently recruiting patients (NCT03878849, advanced ovarian cancer; NCT03562832, metastatic breast cancer).

## Supplementary information

Supplemental Appendix

## Data Availability

The data will not be available for sharing at this time as the data are commercially confidential. However, Eisai will consider written requests to share the data on a case-by-case basis.
