# Supplementary material for: First-in-human study of the PARP/tankyrase inhibitor E7449 in patients with advanced solid tumours and evaluation of a novel drug-response predictor
Source: Br J Cancer. 2020 Jun 11;123(4):525–33. doi: 10.1038/s41416-020-0916-5 (PMC7434893; doi:10.1038/s41416-020-0916-5)
Supplement: Supplementary file 1 — Supplemental Appendix [file 41416_2020_916_MOESM1_ESM.docx]

# Supplemental Appendix

**Supplemental Table 1**. Measured growth inhibition of cell lines in the presence
of E7449 (an 8-day assay).

| **Disease Tissue** | **Cell Line** | **IC_50_ values, μM** |
| --- | --- | --- |
| Breast | T47D | 7.8 |
| Breast | MDA-MB-231 | 25 |
| Breast | Hs578T | 9.6 |
| Breast | HCC1143 | 3.7 |
| Breast | HCC70 | 9.3 |
| Breast | HCC1806 | 1.8 |
| Breast | MDA-MB-436 | 0.19 |
| Breast | MDA-MB-157 | 6.8 |
| Breast | MDA-MB-468 | 2.5 |
| Breast | MDA-MB-453 | 6.2 |
| Breast | MCF-7 | 1.9 |
| Breast | BT-20 | 7.9 |
| Fibrosarcoma | HT-1080 | 0.83 |
| Melanoma | M14 | 6.7 |
| Prostate | DU145 | 1.8 |
| NSCLC | A549 | 2.2 |
| GBM | SF-295 | 2.6 |
| Colon | HCT-116 | 1.7 |
| Colon | HCT-15 | 2.2 |
| HCC | HepG2 | 3.8 |
| HCC | C3A | 2.54 |
| HCC | SNU-423 | 19 |
| HCC | SNU-182 | 5.4 |
| Endometrial | HEC-1 | 1.28 |
| Endometrial | HEC-251 | 1.26 |
| Endometrial | HEC-108 | 5.97 |
| Endometrial | HEC-59 | 2.44 |
| Endometrial | HEC-6 | 1.88 |
| Endometrial | ECC-1 | 0.68 |
| Endometrial | SNG-M | 1.36 |
| Endometrial | HEC-50B | 3.45 |
| Endometrial | SNG-ll | 5.26 |
| Endometrial | KLE | 2.59 |
| Endometrial | HEC-1B | 7.27 |
| Endometrial | HEC-1A | 2.96 |
| Endometrial | HEC-88nu | 1.14 |
| Endometrial | MFE-296 | 0.67 |
| Endometrial | MFE-280 | 0.77 |
| Endometrial | RL95-2 | 2.13 |
| Endometrial | HEC-151 | 1.44 |
| Endometrial | AN3 CA | 1.49 |
| Endometrial | HEC-265 | 2.40 |
| SCLC | NCI-H1694 | 2.47 |
| SCLC | NCI-H209 | 0.62 |
| SCLC | NCI-H146 | 0.93 |
| SCLC | NCI-H211 | 0.48 |
| SCLC | NCI-H524 | 0.69 |
| SCLC | NCI-H82 | 0.47 |
| SCLC | NCI-H446 | 0.61 |
| SCLC | NCI-H69 | 1.33 |
| Promyelocytic leukemia | HL-60 | 10.3 |
| Erythroleukemia from CML patient | K562 | 4.3 |
| B cell lymphoma | SR | 0.26 |
| B lymphoblast | IM-9 | 2.1 |
| NHL B cell lymphoma | RL | 2.5 |
| Histiocytic lymphoma - monocytic | U937 | 2.7 |
| Burkitt's lymphoma | Namalwa | 2.5 |
| Myeloma | MC/CAR | 2.3 |
| Myeloma | NCI-H929 | 5.2 |
| Myeloma | U266 | 6.7 |
| DLBCL (GC) | DB | 1.6 |
| DLBCL (GC) | HT | 0.66 |
| DLBCL (GC) | Pfeiffer | 0.71 |
| DLBCL (GC) | Toledo | 1.6 |
| MCL | Jeko-1 | 1.9 |
| MCL | JVM-13 | 7.3 |
| MCL | JVM-2 | 14 |
| MCL | Mino | 3.4 |
| MCL | NCEB-1 | 5.3 |
| AML | KG-1 | 2.0 |
| AML | THP-1 | 5.4 |
| AML | MV-4-11 | 0.95 |
| Myeloma | RPMI-8226 | 9.3 |
| Myeloma | ARH-77 | 3.2 |

AML, acute myeloid leukemia; CML chronic myelogenous leukemia; DLBCL, diffuse large B-cell lymphoma; GBM, glioblastoma; HCC, hepatocellular carcinoma; MCL, mantle cell lymphoma; NHL, non-Hodgkin lymphoma; NSCLC, non-small-cell lung carcinoma; SCLC, small cell lung cancer.

### Supplemental Table 2. Genes in the drug-response predictor biomarker profile that are associated with DNA damage response or Wnt/β-catenin pathways.

| Pathway | Genes associated |
| --- | --- |
| Wnt/β-catenin | RAPGEF5, PRPF4, CUL3, CHD4, HNRNPM, HNRNPU, SRSF1, STRAP, DDX39A, UBE2S, TCF4, ZNF24, BOP1, DKC1, FAM134B, ACLY, UBE2M, MTF2, EWSR1, SKP2, HNRNPD, ILKAP, PKN1, FUS, NCL, ANP32A, STIP1, FAM134B, BCL11A, SOX4, MAPRE2, RFC3, PBK, KHDRBS1, SSRP1, BHLHE40, S100A11, STAT1, VEGFA, IRF1, LMNA, PIEZO1, LGALS3, LDLRAP1, CFLAR, ZFP36, CBX7, RARRES3, IQGAP1, IL6ST, CNN2, ISG15, CDH11, ABCC3, NNMT, SPTBN1, ITGA5, RAC2, S100A4, ALDOA, PLAC8, USP4, DUSP5, CYR61, ACSL5, CTNND1, GPX4, FN1, COL1A1, FOS, CYLD, GATA6, CCND1, CYP1B1, FLII, STAT6, MYOF, AXL, GATA3 |
| Base excision repair | PNKP |
| Nucleotide excision repair | RFC3, RPA1, CUL3 |
| Mismatch repair | MSH2, RPA1, RFC3 |
| Homologous repair | SRSF3, RUVBL1, RPA1, RFC3, CHD4, PRPF8, RBMX, ACLY, EWSR1, HNRNPD, MAP3K7, SFPQ, SSRP1, LGALS3, SPTBN1, CCND1 |
| Non-homologous end-joining | PNKP, WWP2 |
| Translesion synthesis | ISG15 |
| Other DNA damage response | SRSF1, SRSF7, PRMT1, PRMT5, SOX4, YWHAB, BUB1B, E2F5, STRAP, UBE2S, DESI2, GAR1, DKC1, DNAJC7, RRP1B, UBE2M, SKP2, FUS, NCL, SAFB, PBK, PHB, KHDRBS1, GADD45B, S100A11, IRF1, CD59, SUN2, INPP4B, DUSP1, IER3, ZFP36L1, IFI16, MVP, EIF1, DUSP5, CYR61, FOS, CYLD, STAT6, AXL |

**Supplemental Table 3**. Effect of food on E7449 pharmacokinetic parameters (pharmacokinetic analysis set).

| **Pharmacokinetic Parameter** | **E7449 600-mg Dose** | |
| --- | --- | --- |
|  | **Fed (n=11)** | **Fasted (n=10)** |
| C_max_ (ng/mL) | 863 (563) | 1470 (697) |
| t_max_ (h) | 4.05 (1.07, 8.18) | 2.01 (0.97, 3.00) |
| AUC_(0-24h)_ (ng·h/mL) | 6150 (3280) | 5510 (1730)  (n=9) |
| AUC_(0-t)_ (ng·h/mL) | 6150 (3270) | 5310 (1780)  (n=10) |
| AUC_(0-inf)_ (ng·h/mL) | 6600 (1430)  (n=5) | 4480 (1850)  (n=2) |
| t_1/2_ (h) | 5.64 (3.17)  (n=5) | 6.11 (0.74)  (n=2) |
| C_av,ss_/F (ng/mL) | 276 (130)  (n=8) | 229 (72.1)  (n=9) |
| CL_ss_/F (L/h) | 131 (119)  (n=8) | 124 (55.0)  (n=9) |
| R_ac_ | 1.08 (0.12)  (n=5) | 1.07 (0.03)  (n=2) |

Data are the mean (standard deviation) except for t_max_.

For t_max_, the median (minimum – maximum) are shown.

AUC_(0-24)_, area under the concentration–time curve from time zero (predose) to 24 hours postdose; AUC_(0-t)_, area under the concentration–time curve from time zero (predose) to time of last quantifiable concentration; AUC_(0-inf)_, area under the concentration–time curve from time zero (predose) extrapolated to infinite time; C_av,ss_, average steady-state concentration during multiple-dose administration; C_max_, maximum observed concentration; CL_ss_/F, apparent total clearance at steady state following oral administration; R_ac_, accumulation ratio; t_max_, time at which the highest drug concentration occurs; t_1/2_, terminal elimination phase half-life.

### Supplemental Table 4. Pharmacokinetic parameters estimated by compartmental model analysis following oral administration of E7449 and pharmacodynamic parameters estimated for the indirect response model fit of the inhibition of PAR formation by E7449.

| **Pharmacokinetic parameters** | | | **Pharmacodynamic parameters** | | |
| --- | --- | --- | --- | --- | --- |
| **Parameter** | **Estimate** | **%Coefficient of Variation** | **Parameter** | **Estimate** | **%Coefficient of Variation** |
| k_a_ (h^-1^) | 0.82 | 17.6 | I_max_ | 1 | fixed |
| V/F (L) | 149.7 | 30.9 | EC_50_ (µg/mL) | 0.11 | 26.1 |
| k_e_ (h^-1^) | 0.51 | 35.9 | k_in_ (h^-1^) | 35.5 | 17.9 |
| k_12_ (h^-1^) | 0.21 | 47 | k_out_ (h^-1^) | 0.52 | 14.4 |
| k_21_ (h^-1^) | 0.27 | 10.4 | gam | 1 | fixed |

EC_50_, concentration of drug at which 50% of the maximum effect is produced; gam, the sigmoidicity factor (Hill coefficient); I_max_, the maximal inhibitory effect (percent inhibition from baseline); k_a_, absorption rate constant; k_e_, first-order elimination rate constant; k_in_, uptake rate constant; k_out_, elimination rate constant; k_12_, transfer rate constant (first-order) from the central (1) to peripheral (2) compartment; k_21,_ transfer rate constant (first-order) from the peripheral (2) to central (1) compartment; V/F, volume of distribution for the central compartment.

**Supplemental Table 5**. *BRCA-*mutated patients: prior platinum therapy and best overall response to E7449.

| **Prior Platinum Therapy** | **Tumor Site** | **Best Overall Response to E7449** |
| --- | --- | --- |
| Naïve | Breast | Progressive Disease |
| Resistant | Breast | Progressive Disease |
| Naïve | Pancreas | Stable Disease |
| Sensitive | Ovary | Stable Disease |
| Sensitive | Ovary | Partial Response |
| Sensitive | Ovary | Progressive Disease |

### Supplemental Figure 1. A Plot of mean (± standard deviation) percentage change from baseline PAR by dose (pharmacodynamic analysis set). B Mean PAR over time superimposed on E7449 plasma concentration (pharmacokinetic analysis set; all doses).


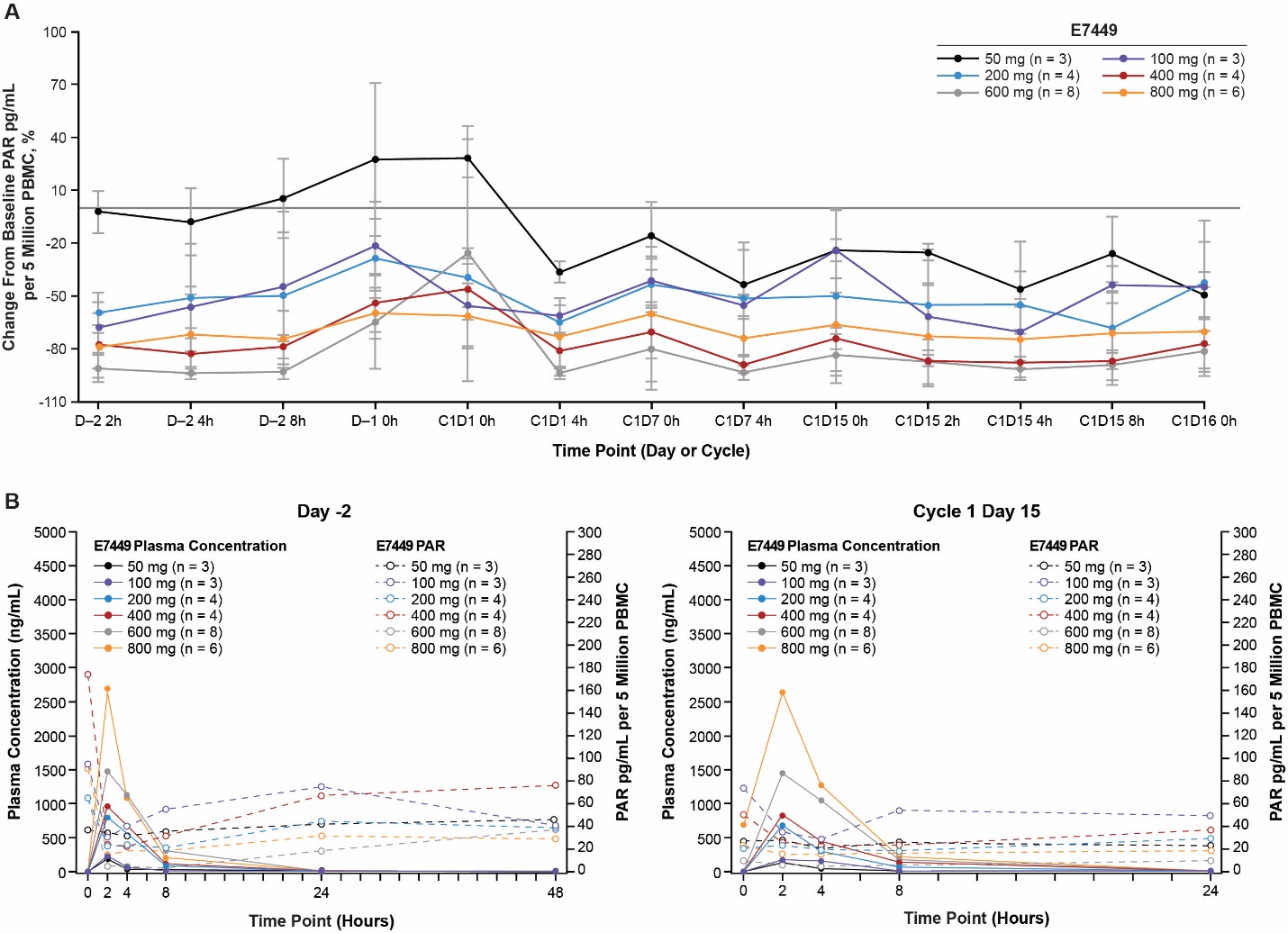


C#, cycle number; D#, day number; PAR, polyadenosine diphosphate-ribose; PBMC, peripheral blood mononuclear cells.

**Supplemental Figure 2.** Mean PAR and E7449 plasma concentration over time (600-mg dose; food-effect cohort). Mean PAR levels are low at time 0 in the fasted (n=10) and fed (n=11) groups as patients received E7449 either with or without food after an overnight fast on cycle 1, day 7. The patients randomized to the fed condition received E7449 immediately after consuming a high-fat meal.


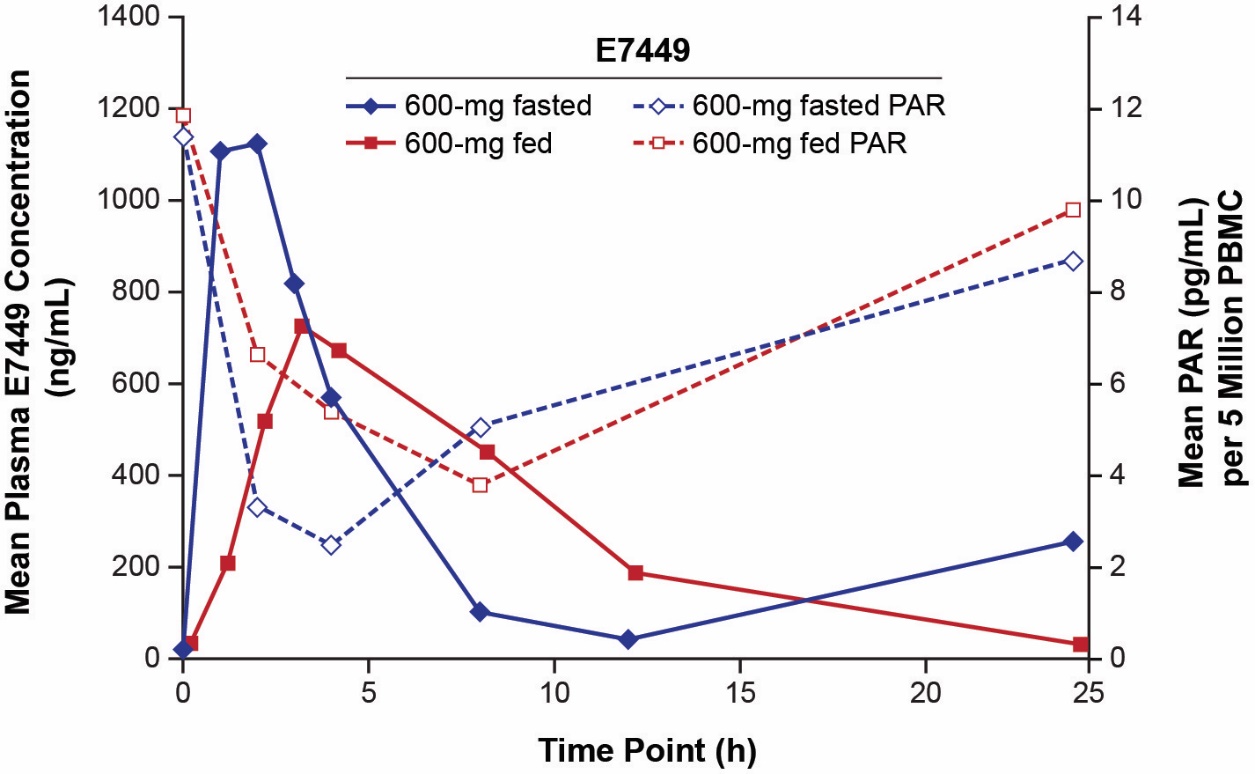


PAR, poly adenosine diphosphate-ribose; PBMC, peripheral blood mononuclear cells.

**Supplemental Figure 3**. Pharmacokinetic/pharmacodynamic indirect-response model. In **A**, symbols represent mean concentrations, lines are the fitted curves, and the error bars represent standard deviation of mean observed E7449 plasma concentrations. In **B**, symbols represent the observed mean percentage PAR response, lines are the fitted curves obtained by the pharmacokinetic/pharmacodynamic model, and error bars represent standard deviation of the mean percentage PAR response. In **C**, simulations were conducted with the pharmacokinetic and pharmacodynamic parameters indirect-response model.


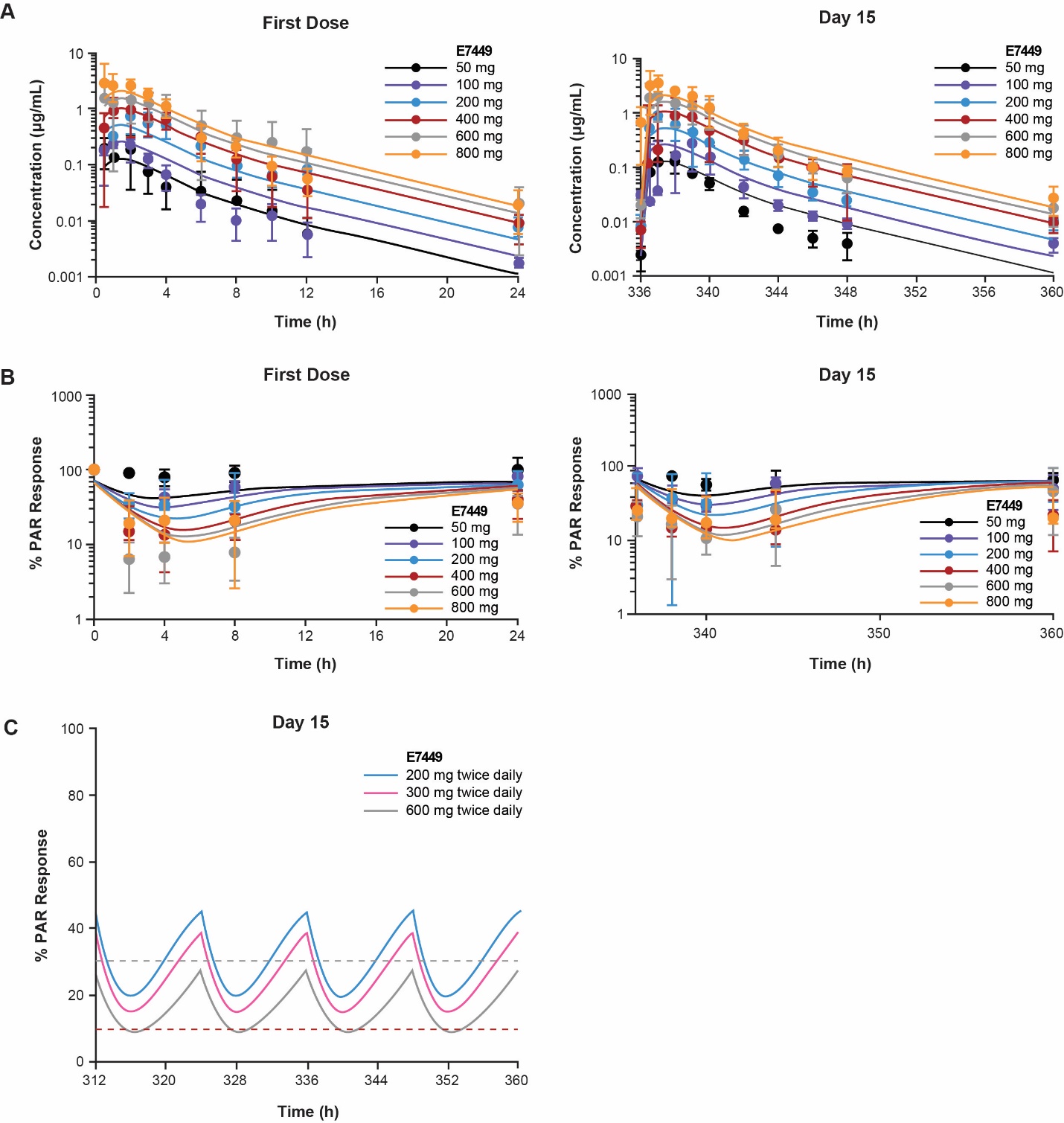


PAR, polyadenosine diphosphate-ribose.

**Supplemental Figure 4**. Percentage change from baseline in total sum of target lesion diameters.


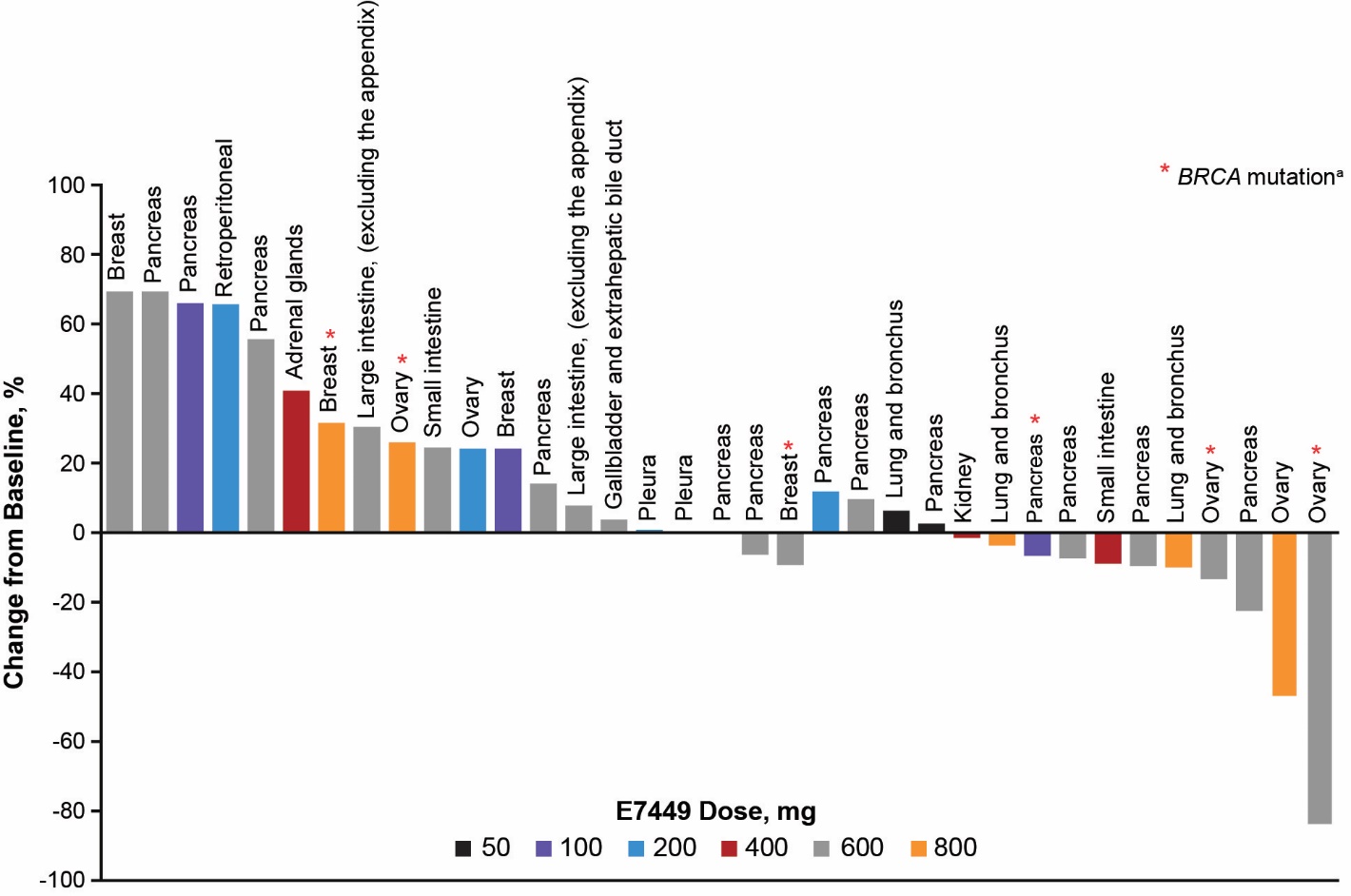


*^a^BRCA* mutation status was obtained from the investigator and was not a central assessment in the trial.

**Supplemental Figure 5**. Serum soluble IL-2Rα changes over time. Lines represent individual patient serum samples available from each dose cohort **A**. The mean of 2 baseline samples (days −3 and −2 prior to dosing) was used to calculate percent change from baseline at each of the postdosing time points. Serum samples collected from healthy donors at days 0, 3, 7, and 14 were tested to assess endogenous variability **B**. Dotted line shows +/− 20% change from baseline.


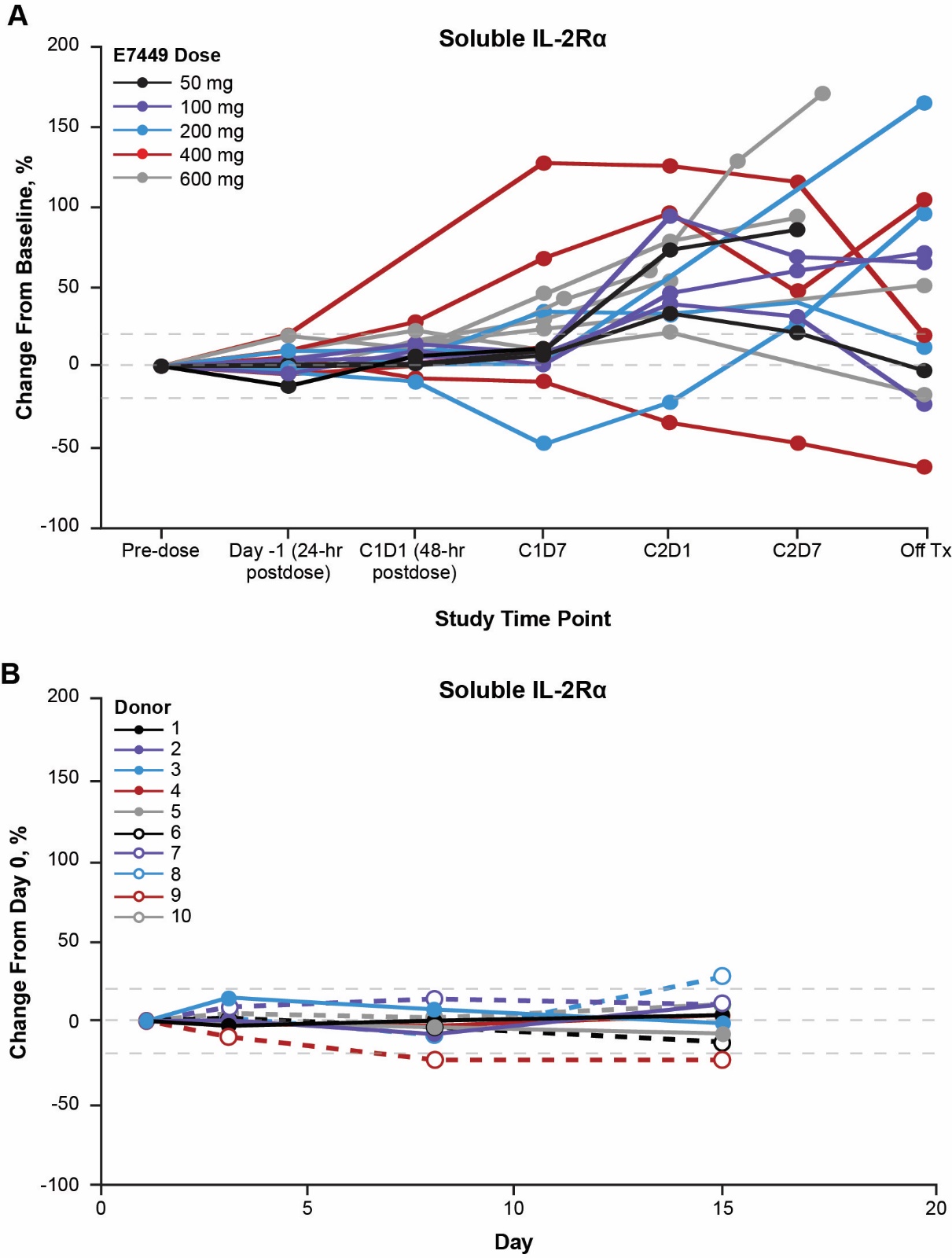


C#, cycle#; D#, day#; IL-2Rα, interleukin-2 receptor α; Tx, treatment.
